# Risk factors for hypoglycemia in patients with type 2 diabetes mellitus after intensive insulin therapy and blood glucose monitoring strategy

**DOI:** 10.4314/ahs.v23i2.58

**Published:** 2023-06

**Authors:** Ling Cai, Weiping Wang, Lian Dai

**Affiliations:** 1 Department of Outpatient, Nanjing First Hospital, Nanjing Medical University, Nanjing 210006, Jiangsu Province, China; 2 Department of Endocrinology, Nanjing First Hospital, Nanjing Medical University, Nanjing 210006, Jiangsu Province, China; 3 Department of Nursing, Nanjing First Hospital, Nanjing Medical University, Nanjing 210006, Jiangsu Province, China

**Keywords:** Diabetes mellitus, glucose monitoring, hypoglycemia, insulin therapy, risk factor

## Abstract

**Background:**

To explore the risk factors for hypoglycemia in patients with type 2 diabetes mellitus (T2DM) after intensive insulin therapy and the blood glucose monitoring strategy.

**Methods:**

A total of 172 T2DM patients diagnosed from March 2019 to March 2021 were randomly divided into training (n=115) and test sets (n=57), and given intensive insulin therapy. After treatment, the training set was divided into hypoglycemia (n=35) and non-hypoglycemia groups (n=80). Univariate and multivariate logistic regression analyses were performed. Then 120 patients were divided into instantaneous scanning glucose test system monitoring (observation) and glucometer monitoring (control) groups. The diagnostic values of the two methods were validated using receiver operator characteristic curves.

**Results:**

Course of disease, body mass index, fasting C-peptide and creatinine were independent risk factors for hypoglycemia, while instantaneous scanning glucose test system monitoring and glucometer monitoring were protective factors (P<0.05). The model had high predictive value. The observation group had shorter time of blood glucose reaching the standard, smaller insulin dose, and lower risk of hypoglycemia than those of the control group (P<0.05).

**Conclusion:**

During intensive insulin therapy by multiple subcutaneous injections, the clinical effect of instantaneous scanning glucose test system on T2DM patients is better than that of glucometer.

## Introduction

Diabetes mellitus is one of the main non-infectious chronic diseases threatening human health nowadays, of which type 2 diabetes mellitus (T2DM) accounts for more than 90%^1^. Currently, intensive insulin therapy is an effective treatment method for T2DM. However, an insulin pump is needed to continuously control blood glucose, and patients are prone to a variety of complications after treatment. Among them, the most common complication is hypoglycemia^2^. Hypoglycemia is a common acute complication of diabetes mellitus and the main obstacle to glycemic control. Particularly, the patients receiving intensive insulin therapy during hospitalization are prone to hypoglycemia which increases the incidence rates of cardiovascular, cerebrovascular diseases and neurological dysfunction, resulting in falls, fractures, convulsions and even coma^3^. With the continuous decline of pancreatic islet function, the course of T2DM is prolonged, and the risk of hypoglycemia is also elevated^4^. In addition to the influence of drugs and insulin dose, hypoglycemia is also related to the individual differences of patients, mainly including age, course of disease, type of accompanying chronic diseases and severity of complications^5^. Therefore, it is necessary to explore the risk factors for hypoglycemia after treatment, and to improve the blood glucose monitoring methods.

In the present study, the clinical data of 172 T2DM patients undergoing intensive insulin therapy were retrospectively analysed, and the incidence of hypoglycemia after treatment and its risk factors were explored. To better monitor the blood glucose during treatment, the patients were grouped, and the value of instantaneous scanning glucose test system for blood glucose monitoring was assessed. The aim of this study was to provide valuable clinical evidence for preventing hypoglycemia in patients with T2DM after intensive insulin therapy.

## Materials and Methods

### General data

A total of 172 patients diagnosed as T2DM in our hospital from March 2019 to March 2021 were selected as the subjects, including 93 males and 79 females aged 4570 years old, with an average of (59.32±11.42) years old. The course of disease was 6 months to 12 years, with an average of (4.69±1.53) years. The body mass index (BMI) was (22.36±6.65) kg/m2. The patients were given intensive insulin therapy using insulin pumps (Medtronic, USA; model: 712). Instantaneous scanning glucose test system was used for 102 patients, while glucometer was used to detect the fingertip blood glucose for 90 patients. This study was approved by the ethics committee of our hospital, and all patients enrolled and their families were informed of this study and signed the informed consent.

### Diagnostic criteria

T2DM: (1) Fasting blood glucose >7.0 mmol/L, (2) 2-h postprandial blood glucose >11.1 mmol/L, and (3) glycated hemoglobin >6.5%.

Hypoglycemia: After treatment, the peripheral blood glucose levels after every meal and before sleep were monitored using the continuous glucose monitoring system, and the hypoglycemic reactions (sweating, palpitation, sense of hunger and fatigue) were observed. Hypoglycemia was diagnosed when peripheral blood glucose or fasting blood glucose <3.9 mmol/L, and 2-h postprandial blood glucose <4.4 mmol/L.

### Inclusion criteria

All patients met the 1999 WHO diagnostic criteria for diabetes mellitus^6^, and all underwent intensive insulin therapy for the first time.

### Exclusion criteria

The patients with ketoacidosis, severe cardiac insufficiency, hepatic-renal dysfunction, or severe infection were excluded.

### Treatment methods

The patients were injected with short-acting insulin twice in the morning and evening (subcutaneous injection before meal + subcutaneous injection before sleep), and also given oral hypoglycemic drugs. The initial dose of insulin was determined according to the patient's body weight on admission, and its dose was adjusted based on the monitored blood glucose level. The therapy lasted for a total of 14 d.

### Instantaneous scanning glucose test system monitoring

For the observation group, the blood glucose was monitored using the instantaneous scanning glucose test system from 24 h before enrolment: The dorsal skin of the upper arm was routinely disinfected with ethanol, into which the sensor was aseptically placed and turned on. The scanner was started to automatically measure the blood glucose level once every 15 min.

### Glucometer monitoring

A conventional glucometer was used to monitor the fasting and 2-h postprandial blood glucose levels, and to guide the intervention. At the same time, the patients wore the instantaneous scanning glucose test system to collect the research data later (researchers were single-blinded to the results during monitoring).

### Observation indices

The venous blood glucose levels on an empty stomach before treatment, and at 30 min, 1 h, 2 h and 3 h after meal were measured. At 12-14 d before and after treatment, the fingertip blood glucose was also measured before and after every meal and before sleep.

### Grouping of training and test sets

All patients underwent intensive insulin therapy by multiple subcutaneous injections. Then they were divided into training set (n=115) and test set (n=57) at a ratio of 3:1 using the computer-generated random number method (Table 1).

**Table 1:** General data of training and test sets

Group	Male [n(%)]	Height (m)	Northerner [n(%)]
Training set (n=115)	62 (53.91)	1.67±2.14	43 (37.39)
Test set (n=57)	31 (54.39)	1.63±1.27	21 (36.84)
χ^2^/*t*	0.003	0.109	0.005
P	>0.05	>0.05	>0.05

### Statistical analysis

SPSS 23.0 software was used for statistical analysis. Measurement data were expressed as mean ± standard deviation ( x̅ ± s) , and compared between two groups by the t test. Count data were expressed as percentage (%), and compared between two groups by the χ2 test. The risk factors for hypoglycemia in T2DM patients after intensive insulin therapy were explored through multivariate logistic regression analysis. P<0.05 was considered to be statistically significant.

## Results

### Incidence of hypoglycemia after intensive insulin therapy

Hypoglycemia occurred in 35 cases (30.43%) in the training set for 62 times in total, including 34 times of asymptomatic hypoglycemia. The incidence rates of hypoglycemia before every meal, after every meal and before sleep were 61.29%, 27.43% and 37.10%, respectively (Table 2).

**Table 2:** Incidence time, frequency and composition ratio of hypoglycemia

Time	Frequency	Composition ratio
Before breakfast	16	25.81%
After breakfast	7	11.29%
Before lunch	12	19.35%
After lunch	4	6.45%
Before dinner	10	16.13%
After dinner	6	9.68%
Before sleep	23	37.10%

### Univariate analysis results of hypoglycemia occurrence

After treatment, the training set was divided into hypoglycemia group (n=35) and non-hypoglycemia group (n=80) according to whether hypoglycemia occurred, and their general data were compared. The hypoglycemia group had lower BMI and fasting C-peptide level, longer course of disease, time of blood glucose reaching the standard and length of stay, and higher creatinine level and proportion of complications such as coronary heart disease than those of the non-hypoglycemia group. In addition, the blood glucose monitoring method was also an influencing factor for hypoglycemia after treatment (P<0.05). Age, obesity, hypertension, peripheral vascular disease and diabetic neuropathy had no significant differences between the two groups (P>0.05) (Table 3).

**Table 3:** Univariate analysis results of hypoglycemia after treatment [n (%)]

Item	Hypoglycemia group (n=35)	Non-hypoglycemia group (n=80)	χ^2^/*t*	P
Age (Y)	59.36±8.46	51.28±5.29	0.633^a^	0.528
Course of disease (Y)	7.35 ±2.14	5.27 ±1.58	5.808^a^	<0.001
BMI (kg/m*^2^*)	21.46±2.86	25.68±2.39	8.196^a^	<0.001
Time of blood glucose reaching the standard (h)	11.25±4.27	8.98 ±4.36	2.585^a^	0.011
Length of stay (d)	15.16±4.35	10.56±4.62	4.999^a^	<0.001
Chronic diabetic nephropathy	11(31.43%)	17(15.00%)	4.107	0.043
Peripheral vascular disease	13(37.14%)	21(26.25%)	1.387	0.239
Coronary heart disease	15(42.86%)	18(22.50%)	4.931	0.023
Diabetic neuropathy	10(28.57%)	22(27.50%)	0.014	0.906
Hypertension	11(31.43%)	21(26.25%)	0.325	0.509
Lipid metabolism disorders	13(37.14%)	15(18.75%)	4.472	0.034
Obesity	12(34.29%)	16(20.00%)	2.698	0.100
Fasting C-peptide	0.25±0.21	0.58 ±0.16	9.224^a^	<0.001
Creatinine	125.38±22.43	76.48±32.45	8.099^a^	<0.001
Monitoring method			13.004	<0.001
Glucometer	17(48.57%)	43(53.75%)		
Instantaneous scanning glucose test system	18(51.43%)	37(46.25%)		

a *t* value, others: χ^2^ value.

### Multivariate analysis results of hypoglycemia occurrence

The statistically different indices in univariate analysis were subjected to multivariate logistic regression analysis, with whether hypoglycemia occurred after treatment as the dependent variable. The results showed that the course of disease, BMI, fasting C-peptide and creatinine were independent risk factors for hypoglycemia after intensive insulin therapy (P<0.05), while instantaneous scanning glucose test system monitoring and glucometer monitoring were protective factors (P<0.05) (Table 4).

**Table 4:** Multivariate analysis results of hypoglycemia after treatment

Variable	Regression coefficient	Standard error	Wald χ^2^	*OR*	95%*CI*	P
Course of disease (Y)	1.89	0.632	8.859	6.62	1.092~12.523	0.021
BMI (kg/m^2^)	1.67	0.593	7.931	5.31	1.138~10.691	0.032
Fasting C-peptide (ng/mL)	1.463	0.651	5.05	4.32	1.089~8.963	0.026
Creatinine (µmoI/L)	1.666	0.587	8.055	5.29	1.327~11.394	0.017
Glucometer	-0.396	0.627	0.399	0.673	0.018~10.435	0.004
Instantaneous scanning glucose test system	-2.096	0.586	12.750	0.123	0.037~9.843	<0.001

### Prediction model for hypoglycemia after treatment

**Figure 1:**
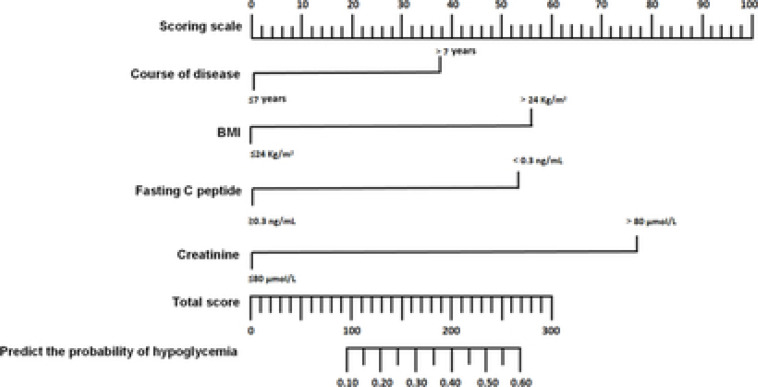
Nomogram prediction model for hypoglycemia in T2DM patients after intensive insulin therapy

The nomogram prediction model for hypoglycemia after treatment was established with independent risk factors. The scores of the course of T2DM, BMI, fasting C-peptide and creatinine were 37.95, 56.21, 53.35 and 77.36 points, respectively, and the total score was 224.87 points. Therefore, the corresponding incidence rate of hypoglycemia was 49.25% (Figure 1).

### Model validation

**Figure 2:**
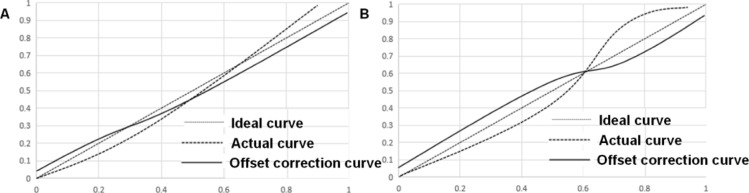
Calibration of nomogram model for training set (A) and test set (B)

**Figure 3:**
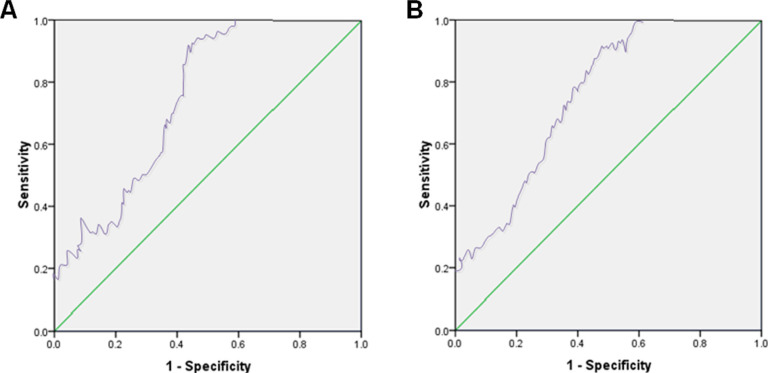
ROC curves of training set (A) and test set (B)

The calibration and efficiency of the established nomogram model were assessed. The C-index values of training and test sets were 0.936 (95%CI: 0.893-0.965) and 0.945 (95%CI: 0.912-0.972), respectively. The actual calibration curve fitted well with the ideal curve, indicating that the conformance of the nomogram model for predicting the risk of hypoglycemia after treatment was good (Figure 2). The receiver operator characteristic (ROC) curve analysis revealed that the areas under the curves (AUC) of the nomogram model of training and test sets were 0.843 and 0.832, respectively, suggesting that the nomogram model had a high predictive value for hypoglycemia after intensive therapy (Figure 3).

### Venous and fingertip blood glucose levels before and after treatment using two monitoring methods

To assess the value of the two protective factors for blood glucose monitoring after treatment, 120 patients were selected and divided into instantaneous scanning glucose test system monitoring group (observation group) and glucometer monitoring group (control group). The two groups had similar venous blood glucose levels on an empty stomach before treatment, and at 30 min, 1 h, 2 h and 3 h after meal (P>0.05) (Table 5). At 12-14 d before and after treatment, the two groups also had similar fingertip blood glucose levels before and after every meal and before sleep (P>0.05) (Table 6).

**Table 5:** Venous blood glucose levels before and after treatment [(x̅ ± s), mmol/L]

	Group (n)	Fasting blood glucose	30 min	1 h	2 h	3 h
Before treatment	Observation group (n=72)	10.34±5.37	11.40±4.93	12.37±4.39	13.21±4.79	13.52±4.73
	Control group (n=48)	10.82±5.48	12.62.±42.54	12.86±52.12	13.16±4.12	13.48±4.92
	P	0.635	0.191	0.576	0.949	0.964
After treatment	Observation group (n=72)	5.21±1.37	6.57±1.02	7.75±0.74	8.24±1.21	7.18±0.56
	Control group (n=48)	5.34±0.83	6.46±0.96	7.92±0.67	8.17±1.88	7.23±0.68
	P	0.557	0.555	0.203	0.804	0.661

**Table 6:** Fingertip blood glucose levels before and after treatment [(x̅ ± s), mmol/L]

	Group (n)	Fasting blood glucose	2 h after breakfast	Before lunch	2 h after lunch	Before dinner	2 h after dinner	Before sleep
Before treatment	Observation group (n=72)	9.86±5.29	10.45±4.64	9.93±4.03	11.22±4.36	9.22±4.36	10.82±5.14	9.76±4.28
	Control group (n=48)	9.42±5.38	11.67±4.73	9.54±3.89	11.18±4.52	9.18±4.81	11.03±4.92	9.85±4.79
	P	0.658	0.164	0.599	0.961	0.962	0.824	0.915
After treatment	Observation group (n=72)	5.02±0.87	6.21±1.13	5.13±1.15	6.16±0.54	5.06±0.59	6.33±0.33	5.24±0.31
	Control group (n=48)	5.14±0.62	6.26±0.98	5.27±1.38	6.23±0.67	5.13±0.56	6.37±0.48	5.31±0.36
	P	0.411	0.803	0.548	0.529	0.517	0.589	0.861

### Time of blood glucose reaching the standard, dosage of insulin and blood glucose fluctuation after treatment

At 14 d after intensive insulin therapy, the time of blood glucose reaching the standard was shorter, the dose of insulin was smaller, and the risk of hypoglycemia after treatment was lower in the observation group than those in the control group (P<0.05) (Table 7). Hence, the instantaneous scanning glucose test system had higher monitoring value than that of glucometer.

**Table 7:** Time of blood glucose reaching the standard, dosage of insulin and blood glucose fluctuation after treatment (x̅ ± s)

Group (n)	Time of blood glucose reaching the standard (d)	Dosage of insulin [U/(kg-d)]	Hypoglycemia per capita (times)	Mean of 24-h blood glucose (mmol/L)	Standard deviation of 24-h blood glucose (mmol/L)
Observation group (n=72)	5.35±0.83	24.27±4.26	0.28±0.54	6.87±1.13	1.72±1.23
Control group (n=48)	6.76±0.93	32.43±5.17	3.12±1.24	9.63±2.46	3.41±1.07
P	<0.001	<0.001	<0.001	<0.001	<0.001

### Diagnostic efficiencies of two methods assessed by ROC curves

**Figure 4:**
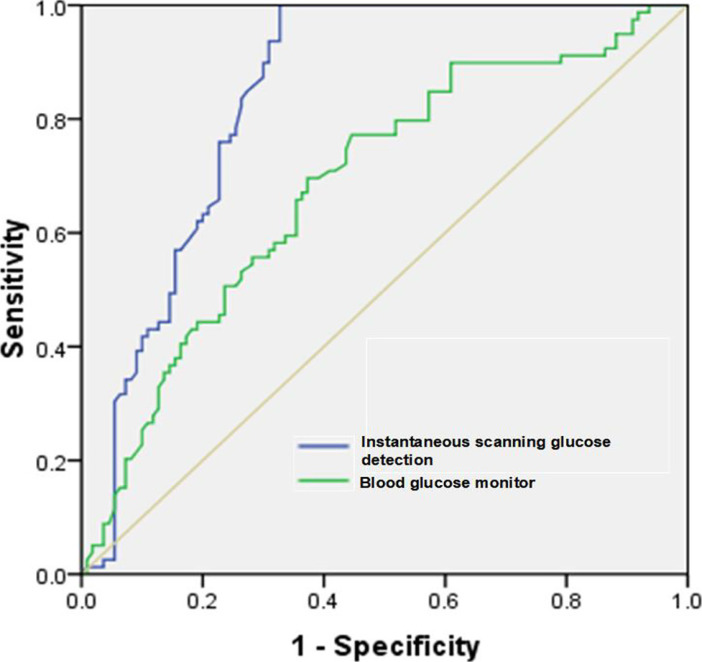
ROC curves of instantaneous scanning glucose test system monitoring and glucometer monitoring

To further assess the monitoring value of the instantaneous scanning glucose test system for hypoglycemia in T2DM patients after intensive insulin therapy, the FSM mathematical model was established, and its diagnostic efficiency was assessed using the ROC curve. The results showed that AUC and sensitivity of instantaneous scanning glucose test system monitoring were 0.87 and 83.25%, respectively, indicating higher diagnostic value. Additionally, the two methods had significantly different specificities (P<0.05) (Figure 4 and Table 8).

**Table 8:** Diagnostic efficiencies of instantaneous scanning glucose test system monitoring and glucometer monitoring

Index	AUC (95%*CI*)	YI	Threshold	Sen (%)	Spe (%)	DA (%)
Glucometer	0.81(0.77-0.91)	0.53	52.36	79.67%	72.83%	77.49%
Instantaneous scanning glucose test system	0.87(0.79-0.93)	0.69	21.94	83.25%	81.33%	86.57%

## Discussion

Diabetes mellitus, as one of the main causes of death and disability globally, occurs when the pancreas cannot produce sufficient insulin or hyperglycemia is caused by insulin resistance. About 425 million people were diagnosed as diabetes mellitus in 2017 around the world, and this number is expected to rise by 50% by 2035. As one of the three types of diabetes mellitus currently, T2DM accounts for 85-95% of all cases. Insulin resistance and/or insulin secretion impairment usually occurs in T2DM patients^7^-^12^. In recent years, intensive insulin therapy has been widely applied in clinical treatment. It is a special method of administration simulating the physiological secretion of pancreatic islet P cells, and the dose of insulin is adjusted according to the patient's physical activity and total sugar intake, so the blood glucose level can return to normal within a short period of time. Intensive insulin therapy can quickly improve the blood glucose index to exert significant therapeutic effects. A variety of side reactions, however, may occur during treatment, the most common of which is hypoglycemia^13^-^16^. Hypoglycemia hinders the safe and effective control of blood glucose in diabetic patients, and increases the risks of trauma, depression and stroke, harming the rehabilitation and affecting the quality of life. Therefore, it is necessary to explore the risk factors for hypoglycemia after treatment. The increased incidence rate of T2DM results from the interaction between genetic and environmental factors^17^.

Strictly controlling the blood glucose level can delay or prevent the occurrence of T2DM complications, but T2DM patients are susceptible to hypoglycemia during insulin therapy^18^. Hypoglycemia is the main complication of insulin therapy in T2DM patients, which raises the risks of cardiovascular events and cognitive impairment, and seriously threatens their life and safety^19^.

The univariate analysis results of the training set herein showed that age, obesity, hypertension, peripheral vascular disease or diabetic neuropathy had no obvious correlation with the occurrence of hypoglycemia after treatment, whereas lower BMI and fasting C-peptide level, longer course of disease, time of blood glucose reaching the standard and length of stay, higher creatinine level and proportion of complications such as coronary heart disease, and blood glucose monitoring methods were all correlated with the onset of hypoglycemia. Furthermore, multivariate logistic regression analysis revealed that the course of disease, BMI, fasting C-peptide and creatinine were independent risk factors for hypoglycemia after intensive insulin therapy, while blood glucose monitoring means during treatment were protective factors. Hypoglycemia of T2DM patients can be effectively prevented by analysing the risk factors and timely adjusting the treatment methods. Silver et al. found that the T2DM patients prone to nocturnal asymptomatic hypoglycemia suffered from obvious blood glucose fluctuations, and the reduction of HbA1c level indicated an increased risk of hypoglycemia^20^.

Based on the independent risk factors for hypoglycemia in T2DM patients after treatment, the nomogram model was established and its accuracy was validated. The C-index and predictive accuracy of the nomogram mode of training set were 0.936 (95%CI: 0.893-0.965) and 85.69%, respectively, while those of the test set were 0.945 (95%CI: 0.912-0.972) and 86.35%, respectively. Taken together, the model had high discriminability and predictive value for hypoglycemia in T2DM patients after intensive insulin therapy. In addition, to mitigate the adverse effect of hypoglycemia on patients, it is also of significance to monitor the real-time blood glucose changes during intensive insulin therapy. Conventionally, the blood glucose indices of patients are usually monitored with glucometer. Intensive insulin therapy required the measurement of blood glucose more than four times a day to safely and effectively adjust the dose of insulin. However, traditional glucometer monitoring is inconvenient because the blood glucose level needs to be calibrated at the fingertip. Through continuously detecting the glucose level in the interstitial fluid, the instantaneous scanning glucose test system generates a complete glucose profile by the sensor, without needing calibration at the fingertip. Therefore, this system is valuable for monitoring the blood glucose indices of T2DM patients during treatment^21^. In the present study, the time of blood glucose reaching the standard was shorter, the dose of insulin was smaller, and the risk of hypoglycemia after treatment was lower in the observation group. Moreover, the ROC curve analysis revealed that AUC and sensitivity of instantaneous scanning glucose test system monitoring were 0.87 and 83.25%, respectively, exceeding those of glucometer monitoring.

## Conclusion

Course of disease, BMI, fasting C-peptide and creatinine are independent risk factors for hypoglycemia in T2DM patients after intensive insulin therapy. During intensive insulin therapy by multiple subcutaneous injections, instantaneous scanning glucose test system has a better clinical effect on T2DM patients than glucometer, accompanied by high safety.
